# High miR-31-5p expression promotes colon adenocarcinoma progression by targeting TNS1

**DOI:** 10.18632/aging.103096

**Published:** 2020-04-21

**Authors:** Bobin Mi, Qiushi Li, Tong Li, Guohui Liu, Jiayang Sai

**Affiliations:** 1 Department of Orthopedics, Union Hospital, Tongji Medical College, Huazhong University of Science and Technology, Wuhan 430022, China; 2 Department of Cardiology, Beijing Chaoyang Integrative Medicine Emergency Medical Center, Beijing 100029, China; 3 Department of Oncology, The Third Affiliated Hospital, Beijing University of Chinese Medicine, Beijing 100029, China; 4 Surgery Department, Brigham and Women’s Hospital, Harvard Medical School, Boston, MA 02115, USA

**Keywords:** colorectal adenocarcinoma, miR-31-5p, TNS1, immune profiling, tumor microenvironment

## Abstract

Overexpression of the miR-31-5p contributes to tumorigenesis and metastasis in diverse neoplasms. In this study, we evaluated expression of miR-31-5p in patients with colon adenocarcinoma (COAD). We found that miR-31-5p was overexpressed in four cohorts (GSE30454, GSE41655, GSE18392, GSE108153) of COAD patients. Importantly, a LinkedOmics analysis revealed that high miR-31-5p expression was associated with poor overall survival of COAD patients. At total of 133 putative target genes of miR-31-5p were identified from TargetScan, miRDB, and TargetMiner. After integrating the target genes with 1,556 deregulated genes in COAD, 8 were acquired that may be targeted by miR-31-5p and contribute to COAD progression. Among these, tensin 1 (TNS1) showed the greatest prognostic ability in COAD and was strongly correlated with M2 macrophages, regulatory T cells, and other immune cells. These findings indicate that, in COAD, miR-31-5p is a potential prognostic factor that affects immune infiltration by targeting TNS1.

## INTRODUCTION

Colorectal cancer (CRC) is a common malignancy throughout the world. CRC accounts for 8% of all cancer-related deaths [1], and the incidence of CRC is increasing, especially in Asian countries [2]. Colon adenocarcinoma (COAD), which accounts for 90% of all CRCs, is the most common histologic subtype [3]. Despite advances in technology, patients with COAD still have a poor prognosis, especially when metastasis to the lymph nodes or distant organs is present. Therefore, understanding the underlying mechanisms in COAD is important to provide new concepts for novel therapies for advanced COAD [4].

MicroRNAs (miRNAs), a family of noncoding RNAs, are prevalent in multicellular and complex eukaryotes and participate in various physiologic processes of cells, including posttranscriptional regulation of gene expression [5, 6]. In cancer, miRNAs regulate various biologic processes of tumor cells, including proliferation, migration, and apoptosis [7, 8]. Notably, aberrant expression of miRNAs contributes to development and progression of COAD [9]. The strong evidence that dysregulation of miRNAs contributes to COAD pathogenesis provides a rationale for targeting miRNAs in cancer treatment.

Using bioinformatic analysis, miRNAs that exhibit oncogenic activity in carcinogenesis have been identified, including miR-21 [10], miR-155 [11], and miR-142 [12]. Literature indicates that miR-31-5p is overexpressed in diverse tumor types [13], and studies have explored its role in lung and breast cancer metastasis [14, 15]. However, the underlying mechanisms of the role of miR-31-5p in COAD remain unknown. Although it has been postulated that miR-31-5p regulates the WNT and Hippo signaling pathways to promote epithelial regeneration following injury [16, 17], we wanted to investigate its function in COAD. Thus, we aimed to evaluate the prognostic value of miR-31-5p and its putative oncogenic functions in COAD and demonstrate the underlying potential mechanism through data mining.

## RESULTS

### miR-31-5p expression in COAD

**Figure 1:**
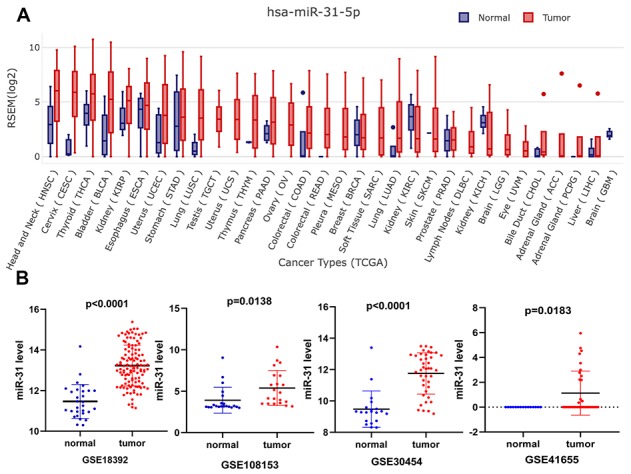
**Expression validation of miR-31-5p.** (**A**) Pancancer expression of miR-31-5p in GEDS. (**B**) Upregulation of miR-31-5p in the microarrays tissues, based on Gene Expression Omnibus (GEO) data sets. Notes: a, GSE18392; b, GSE108153; c, GSE30454; d, GSE41655.

To evaluate the expression level of miR-31-5p in various cancers, we performed a systematic pancancer analysis based on The Cancer Genome Atlas (TCGA) and Genotype-Tissue Expression (GTEx) miRNA databases. The results showed that miR-31-5p is overexpressed in many tumor types, including CRC, breast cancer, lung squamous cell carcinoma, liver hepatocellular carcinoma, and head and neck cancer (Figure 1A). To further validate high expression of miR-31-5p in COAD, microarray data from GEO databases were collected and comparisons between COAD and normal colon tissues were conducted by GEO2R. COAD tissue had significantly higher miR-31-5p expression than the control group tissue in the GSE30454, GSE41655, GSE18392, and GSE108153 data sets (Figure 1B).

### Correlation between miR-31-5p expression and clinical characteristic of COAD patients

**Figure 2:**
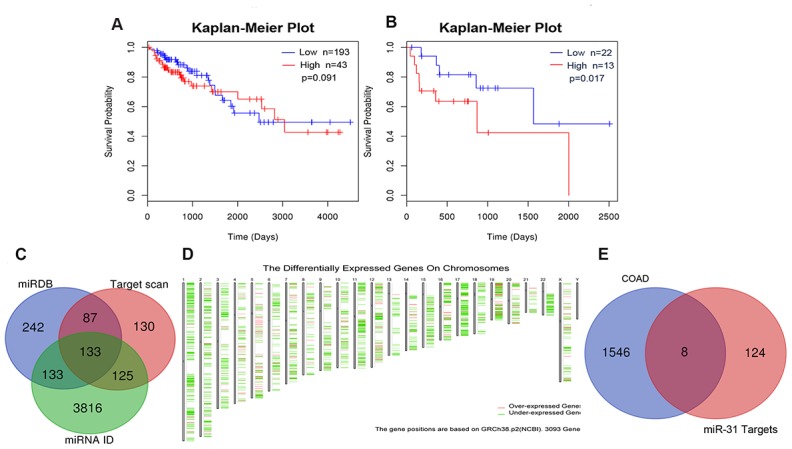
**Prognostic value of miR-31-5p and target genes related with COAD.** (**A**, **B**) Kaplan-Meier curve for miR-31-5p in clinical COAD samples. The *P* values of the Kaplan-Meier curve for COAD patients and pathologic stage IV COAD patients were 0.091 and 0.017, respectively. (**C**) Integration of miR-31-5p predictive genes from TargetScan, miRDB, and TargetMiner. (**D**) The differentially expressed genes in COAD retrieved from GEPIA. The thresholds were set as follows: | Log2 fold change (FC) | ≥ 2 and *P* value<0.01. (**E**) Venn diagram for overlap analysis of miR-31-5p target genes related to COAD.

To assess whether miR-31-5p could be used as a prognostic predictor of COAD, a Kaplan-Meier curve was generated using the LinkedOmics Database. As depicted in Figure 2A, 2B, no statistical difference in survival outcome was observed between COAD patients exhibiting lower and higher expression of miR-31-5p (*P* = 0.091). Because the miR-31-5p expression in COAD patients was significantly different depending on pathologic N stage (*P* = 0.038), we further evaluated the prognostic value of miR-31-5p in different pathologic stages and found that high miR-31-5p expression was significantly correlated with a poor prognosis in patients with pathologic stage IV COAD (*P* = 0.016). In COAD patients with microsatellite stability (MSS) phenotype, miR-31-5p expression was significantly different depending on histologic type (*P* = 5.97^e–4^), number of involved lymph nodes (*P* = 2.5^e–3^), and pathologic stage (*P* = 5.4^e–3^).

### In silico exploration of miR-31-5p targets and their prognostic value

To identify the mechanisms underlying miR-31-5p involvement in different biologic processes of COAD development, the potential targets of miR-31-5p were identified with miRWalk 3.0 target prediction tools. We retrieved target sets from three website servers: TargetScan, miRDB, and TargetMiner. The results of the three predicted target gene sets were integrated by drawing a Venn diagram. One hundred thirty-three overlapping genes were identified as promising targets of miR-31-5p (Figure 2C).

**Figure 3:**
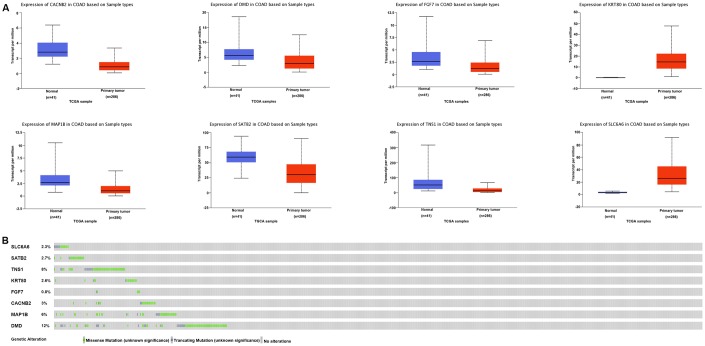
**Expression and mutation analysis of miR-31-5p target genes related to COAD.** (**A**) Expression of integrated genes in COAD and normal tissues based on TCGA samples analyzed by the UALCAN database. (**B**) OncoPrint of integrated gene alterations in COAD. Genomic alterations of the 8 genes are mutually exclusive.

From GEPIA, 1,556 deregulated genes (DEGs) in COAD were identified (Figure 2D). The thresholds of DEGs were set as follows: | Log2 fold change (FC) | ≥ 2 and *P* value < 0.01. After integrating the 133 miR-31-5p target genes with the 1,556 DEGs in COAD, we identified 8 specific genes (SLC6A6, SATB2, TNS1, KRT80, FGF7, CACNB2, MAP1B, and DMD) that might be targeted by miR-31-5p and play a role in COAD progression (Figure 2E). Expression of the 8 genes was evaluated using the UALCAN database. As shown in Figure 3A, 6 genes (SATB2, TNS1, FGF7, CACNB2, MAP1B, and DMD) were underexpressed in tissue samples of COAD patients, which might be related to the overexpression of miR-31-5p in COAD. CBioPortal for Cancer Genomics (cBioPortal, https://www.cbioportal.org/) was applied to explore genetic alterations of these 6 genes. Analysis showed that DMD, TNS1, and MAP1B are the most frequently altered genes with a high ratio of missense mutations based on 619 samples from DFCI COAD data sets (Figure 3B).

### Validation of TNS1 expression and its prognostic value

**Figure 4:**
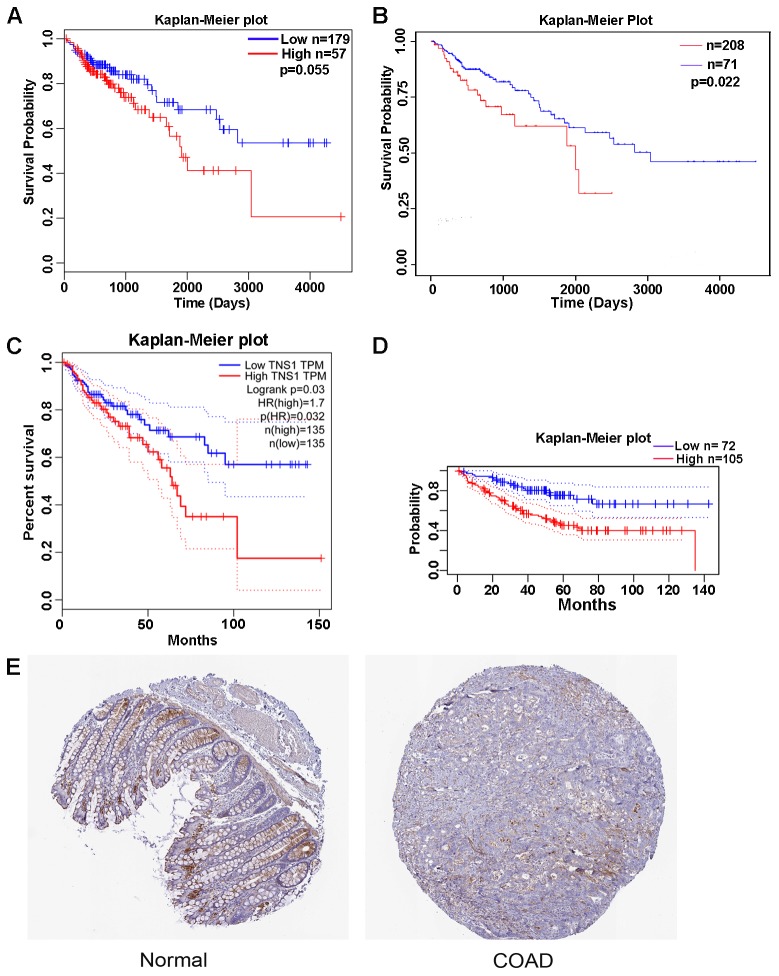
**Kaplan-Meier curve and histochemistry for TNS1 in clinical COAD samples.** (**A**) Kaplan-Meier curve for TNS1 of COAD patients analyzed in the LinkedOmics database. (**B**) Kaplan-Meier curve for TNS1 of COAD patients analyzed in the UALCAN database. (**C**) Kaplan-Meier curve for TNS1 of COAD patients analyzed in GEPIA. (**D**) Kaplan-Meier curve for TNS1 of COAD patients analyzed in the PrognoScan database. (**E**) Histochemistry of TNS1 in normal colon and colorectal adenocarcinoma tissues. The expression distribution of TNS1 in normal colon tissue and CRC patient samples was evaluated in THPA. Normal HPA036089 (ID1857) and tumor HPA036089 (ID 3550) were presented.

To further determine the prognostic value of the 6 genes in COAD, we performed an overall survival (OS) analysis using GEPIA tools, which showed that high expression of TNS1 is correlated with a poor prognosis (*P* = 0.03). Furthermore, the prognostic value of TNS1 was validated using LinkedOmics (*P* = 0.055), the UALCAN database (TCGA samples; *P* = 0.022), and the PrognoScan database (*P* = 0.019; expression histogram: 221747_at) (Figure 4A–4D). Data from LinkedOmics indicated that expression of TNS1 is significantly different depending on number of involved lymph nodes, pathologic N stage, and pathologic stage (*P* = 0.005, 0.011, and 0.048, respectively). TNS1 protein levels in COAD and normal colon tissues were acquired from The Human Protein Atlas (THPA) and are presented in Figure 4E. Distinctly positive TNS1 protein was observed in the epithelium of normal colon tissues, whereas the majority of malignant cells displayed weak cytoplasmic immunoreactivity.

### Correlation of TNS1 expression with immune infiltration level in COAD

**Figure 5:**
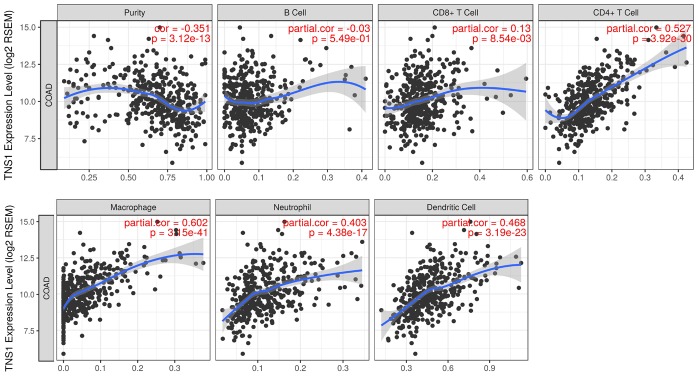
**Correlation of TNS1 expression with immune infiltration level in COAD.** TNS1 expression is significantly correlated with tumor purity and has strong correlations with macrophages, CD4+ T cells, dendritic cells, and neutrophils.

**Figure 6:**
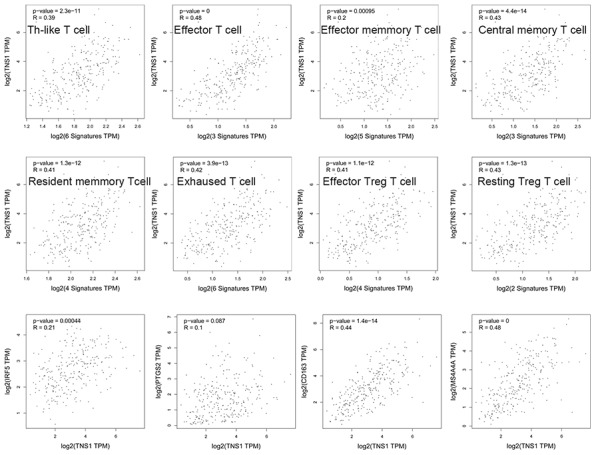
**Correlation of TNS1 expression with immune infiltration level in COAD.** TNS1 is closely related with abundance of T cells and has a stronger relationship with macrophage M2 cells than M1 cells.

We assessed the correlation of TNS1 expression with immune infiltration level in COAD using TIMER. As shown in Figure 5, TNS1 expression was significantly correlated with tumor purity, macrophages (r = 0.602, *P* = 3.15^e–41^), CD4+ T cells (r = 0.527, *P* = 3.92^e–30^), dendritic cells (r = 0.468, *P* = 3.19^e–23^), and neutrophils (r = 0.403, P = 4.38^e–17^). The correlation of TNS1 expression with T cells was further evaluated using GEPIA, which indicated TNS1 is closely associated with naïve T cells (r = 0.43, *P* = 4.3^e–14^), effector T cells (r = 0.48, *P* = 0), central memory T cells (r = 0.43, *P* = 4.4^e–14^), resident memory T cells (r = 0.41, *P* = 1.3^e–12^), exhausted T cells (r = 0.42, *P* = 3.9^e–13^), resting regulatory T (Treg) cells (r = 0.43, *P* = 1.3e^–13^), and effector Treg cells (r = 0.41, *P* = 1.1^e–12^). In addition, correlation of TNS1 with markers of the M1 (PTGS2, IRF5) and M2 (CD163, MS4A4A) phenotypes was analyzed, resulting in correlation indices of 0.1, 0.21, 0.44, and 0.48, with *P* values of 0.087, 0.00, 1.4^e−14^, and 0.00, respectively (Figure 6). The results suggest that TNS1 is more closely related to M2 than M1, indicating TNS1 might regulate macrophage polarization in COAD.

## DISCUSSION

MiR-31 is a significant prognostic predictor in various neoplasms [18] and plays a major role in regulating tumorigenesis in ovarian, breast, lung, and renal cell carcinoma [19–21]. RNA immunoprecipitation results from a microarray study showed that LINC01116 competed with VEGFA to bind with miR-31-5p in tumorigenesis of glioma [22]. Studies have shown that miR-31 expression correlates inversely with metastasis in breast cancer patients, which is achieved via coordinated repression of RhoA [14]. In addition, miR-31 is also involved in immune and inflammation responses, such as regulating T-cell exhaustion during chronic viral infection [23] and acting as a negative regulator of the noncanonical NF-κB pathway in adult T-cell leukemia [24].

The role of miR-31 in COAD has been explored in several studies. In one study, exogenetic overexpression of miR-31 was shown to promote COAD cell growth, invasion, and migration in vitro by repressing its target gene SATB2 [25]. In addition, elevated expression of the long noncoding RNA MIR31HG, the host transcript of miR-31-5p, has been associated with poor prognosis in COAD patients independent of consensus molecular subtypes and cytotoxic T lymphocyte and fibroblast infiltration [26]. Recent studies also revealed that miR-31-5p may be a useful prognostic biomarker for anti-EGFR therapy in CRC because high miR-31-5p expression was associated with shorter progression-free survival [27]. Furthermore, a comprehensive miRNA expression profiling study identified elevated miR-31-5p expression in BRAF-mutant COAD, which highlights its possible functional role in the serrated pathway [28]. In addition, miR-31-5p is also associated with resistance to chemotherapy, such as oxaliplatin [29] and sorafenib [20].

The present study evaluated miR-31-5p expression level in a pancancer analysis and concluded that miR-31-5p is broadly overexpressed in most neoplasms, indicating that miR-31-5p might be a clinically useful biomarker for human malignancies. Our Kaplan-Meier plot revealed that miR-31-5p contributed to tumor progression based on pathologic stage, suggesting miR-31-5p might contribute to tumorigenesis in COAD. In addition, a previous study reported that miR-31-5p is upregulated in all four murine COAD stages and one of the most upregulated miRNAs in the earliest stages, suggesting it may be involved in COAD initiation [30]. Based on out study, miR-31-5p could be involved in both the initiation and metastasis of COAD.

To elucidate the potential mechanism of miR-31-5p involvement in COAD, a computational target prediction was performed, which identified a pivotal miR-31-5p target gene, TNS1, which is downregulated in COAD patients and closely correlated with COAD progression. This is consistent with a recent study that found that TNS1 level was negatively correlated with miR-31 in COAD tumor tissues [31]. TNS1 is a key component of specialized cellular adhesions that bind to extracellular fibronectin fibrils [32]. One study demonstrated that TNS1 was expressed in normal tissues but had greatly reduced expression in tumor tissues [33] and was associated with tumorigenesis. However, it is controversial whether TNS1 plays a negative or positive role in carcinogenesis. Zhou et al. reported that TNS1 was highly expressed in human CRC cell lines SW620 and RKO and promoted CRC cell proliferation and invasion [34]. Zhang et al. reported that miR-548j promoted human breast cancer invasiveness by downregulating TNS1 expression [33]. An in vitro study indicated that higher expression of TNS1 promoted metastasis [35]. Interestingly, we observed that TNS1 was markedly decreased in COAD samples; however, OS analysis showed that high expression of TNS1 was correlated with poor survival outcome. A recent study demonstrated that TNS1 is required for fibronectin fibrillogenesis on extracellular vesicle fractions by promoting clustering of extracellular matrix–bound integrins and that its depletion significantly inhibits pulmonary metastasis [36]. Phospho-TNS1 was highly elevated in EMT cells after TGFβ treatment and was specifically observed in tissue samples of patients with poor-prognosis lung adenocarcinoma [37]. Based on this evidence, TNS1 negatively impacts COAD tumorigenesis and may accelerate metastasis by regulating epithelial to mesenchymal transition (EMT).

Immune infiltrate correlation analysis indicates that TNS1 is strongly correlated with macrophages, which are the most abundant hematopoietic cells in the COAD tumor microenvironment (TME) [38]. We speculate that TNS1 may play an important role in COAD TME. It is believed that M1 and M2 macrophages are active in tumor prevention and tumor promotion, respectively. Our results indicate that TNS1 is more closely associated with M2 macrophages, suggesting TNS1 might negatively affect tumorigenesis of COAD by enhancing M2 polarization. Because a high Treg cell ratio in tumors is associated with poor survival [39], the relationship between TNS1 and Treg cells observed in the present study further validates the involvement of TNS1 in COAD tumorigenesis. In fact, previous reports have indicated that miR-31 can mediate immune reaction. However, whether miR-31 interacts with tumor-infiltrating immune cells by targeting TNS1 and whether miR-31 plays different roles in CRC according to microsatellite instability status warrant further investigation. In addition, although miR-31 has been validated as a pivotal marker involved in COAD, a combination of different markers may provide better prognostic prediction [40, 41].

In summary, the current study confirmed the overexpression of miR-31-5p in COAD. More importantly, miR-31-5p may be a latent tumor biomarker that can serve to predict prognosis in patients with COAD, especially in those with pathologic stage IV disease. Bioinformatics analyses identified TNS1 as a potential gene targeted by miR-31-5p that may regulate immune cell infiltration and play a vital role in TME and tumorigenesis of COAD.

## MATERIALS AND METHODS

### Expression level of miR-31-5p in COAD

Gene Expression Display Server (GEDS, http://bioinfo.life.hust.edu.cn/web/GEDS/) was used to explore the expression pattern of miR-31-5p in COAD [42]. GEDS is a platform that collects 40 tissues and 1,594 cells lines from The Cancer Genome Atlas (TCGA), Genotype-Tissue Expression (GTEx), Cancer Cell Line Encyclopedia (CCLE), and MD Anderson Cell Lines Project (MCLP), providing information on human gene, miRNA, and protein expression in tissues and cell lines. In addition, we collected microarray data from the GEO database (GSE30454, GSE41655, GSE18392, and GSE108153) to compare the expression of miR-31-5p in COAD and normal tissue.

### Analysis of survival and miR-31-5p in COAD

The correlation between miR-31-5p and survival in COAD was analyzed using the LinkedOmics database (http://www.linkedomics.org/) [43]. The LinkedOmics database collected multiomics data and clinical data of 32 cancer types from The Cancer Genome Atlas (TCGA) project. The thresholds were set according to the following steps: Step 1: TCGA_Colorectal adenocarcinoma (COADREAD); Step 2: miRNASeq, HS miR platform; Step 2b: histological_type colonadenocarcinoma [N:391]; Step 3: miR-31-5p; Step 4: TCGA_COADREAD, Clinical data type, clinical platform; and Step 5: Non-Parametric Test (Attribute Dependent).

### Prediction of miR-31-5p targets

To identify the targets of miR-31-5p, we searched the miRNAwalk3.0 website (http://mirwalk.umm.uni-heidelberg.de/), and three databases (TargetScan, miRDB, and TargetMiner) were mined. Only those genes predicted by all three databases were recognized as target genes. Then, we collected differentially expressed genes (DEGs) associated with COAD from the Gene Expression Profiling Interactive Analysis (GEPIA) online database (http://gepia.cancer-pku.cn/). Genes that were both miRNA target genes and COAD-related genes were determined using a Venn diagram.

### Expression and survival analysis of overlapping genes

The expression and methylation of overlapping genes were evaluated using the UALCAN database (http://ualcan.path.uab.edu/index.html) [44]. To evaluate the prognostic value of overlapping genes in COAD, we analyzed the overall survival (OS) rate of those genes in COAD using the GEPIA tool. The thresholds were set as follows: median group cutoff, 95% confidence interval, and *P* = 0.05 significance level. Then, we further validated the OS of those genes in COAD using UALCAN database and PrognoScan (http://dna00.bio.kyutech.ac.jp/PrognoScan/) [45]. In addition, immunohistochemical results of pivotal targeted genes in normal colon tissue and COAD tissue were obtained from The Human Protein Atlas (THPA, https://www.proteinatlas.org/).

### Gene correlation analysis with immune infiltration

The online TIMER database was used to assess the correlation of gene expression with the level of immune infiltrates [46]. The gene module in TIMER allows users to analyze the gene expression with CD4+ T cells, CD8+ T cells, macrophages, dendritic cells, and neutrophils. Furthermore, the gene expression with the T-cell infiltrates in COAD were confirmed using GEPIA.
